# Application of Artificial Teeth, by the Auroplastic Principle

**Published:** 1854-01

**Authors:** Edward Truman

**Affiliations:** Dentist, London


### *Application of Artificial Teeth, by the Auroplastic Principle.*

By EDWIN TRUMAN, Dentist, London.

*The use of Gutta Percha as a base for Artificial Teeth.*—Mr. TRUMAN first describes the usual method of inserting Artificial teeth, and the objections against the various methods now in use, and then introduces the subject of the use of Gutta Percha, as follows:

It appears, from the foregoing remarks, that an entirely new material should, if possible, be found for the construction of the plate, either alone, or in conjunction with those already in use, to relieve the pressure on the soft structures of the mouth; and, at the same time some method should, if possible, be adopted to procure *certainty* in the fit, combined with solidity and durability in the material. The substance used must be pure, and free from any evil or injurious effect on the structures of the mouth—it should not be hard, or rather, not much harder than the gums themselves—it should be capable of resisting the solvent properties of the saliva and acid, and should be easy of manipulation. Such a substance we possess in gutta percha, and I purpose to show the method by which this substance may be used in the construction of artificial dentures, and be made the means of remedying the greatest defects now existing in them, from its possessing many qualities combined that are not to be so found in any other substance. I shall first point out its peculiarities; not that it is a substance now unknown or unappreciated, but because I think I shall show it to possess qualities that eminently fit it for the purpose of the dentist. It has been long in use for filling teeth, and has been used in the construction of false palates, therefore its qualities in the mouth have been well tested, even in its impure state, as until very lately the pure white gutta percha could not be obtained. This I presume to have been the reason why none of my professional brethren have yet introduced it into the manufacture of artificial teeth. I have, since its first appearance in this country, been deeply interested in it, and have tried innumerable experiments with it. It is, at present, little appreciated, compared to what it will be—and I have no hesitation in saying that a tithe of its capabilities are not known, and that it will be turned to uses, compared with which, in importance, its applications hitherto are as nothing. I will now enumerate some of its most important qualities and peculiarities, and show in what way they serve the dentist; and, in conclusion, I shall briefly detail the mode by which I intend to apply its usefulness to my art.

First, as to its purity. This must of course be our first consideration, and in considering this part of our subject, there are many points requiring attention. Is it capable of decomposition, producing an unhealthy influence? Does it become softened and offensive? These questions must be answered before we can apply it to our use. Is it a substance that can be retained in the mouth for a long time without detriment to the health of the patient, or injury to the surrounding tissues?

It is in its pure state one of the most indestructible substances known, and in cases under my own observation, has been worn for years in the mouth without in the slightest injuring either the general health of the patient, or the tissues that come in contact with it. I have used it now many years; and although it does not wear the same in all mouths, yet in none have I found the slightest ill effects. On the contrary, the mouths of patients having gutta percha palates are so little inconvenienced by it, that after it is removed, it is impossible, upon examination, to tell that any artificial work had been there. This is not the case even with bone; and with gold worn for any length of time, a distinct mark is left upon the gum, the whole of the surface covered by the plate being a darker red than the rest. This is, perhaps, no very great defect; but I mention it as a proof of the inoffensive nature of gutta percha, in the use of which substance, it never happens. Bone worn in the mouth sooner or later decays, and in so doing, becomes black, soft and offensive, and must be replaced—the whole structure being useless. When durability therefore is a matter of consequence, gold must be employed instead; as it is in all cases more or less. No two cases fit the mouth equally comfortably and well; and nothing is more annoying, after a set of teeth have become comfortable and the mouth accustomed to them, than to find from decay that they are worn out and a new set requisite, which, like new shoes on tender feet, must be worn some time before they are easy. These defects do not exist with gutta percha; it does not decay in the mouth, and, if it did, it would not be accompanied by the same disagreeable consequences as ivory.

I have had some cases worn nearly three years, that remained the same as in the first hour they were made. Others, by the friction of the mouth and food, become slightly abraded at the edges; but never softened, never inherently offensive. When any offensive odor does exist, it is from foul breath, want of cleanliness, or from diseased stumps, which the patient has refused to have extracted, (an operation, it is not in the least necessary to undergo, when gutta percha is used.) Experience, therefore, has fully answered the question of its purity. There is, however, one more advantage: even should it wear away, it does not spoil the case, since it can be replaced at one sitting, at any time, and that without in any way altering the old comfortable artificial teeth to which the mouth has become accustomed, and this can be repeated to any extent.

Next, its *specific gravity* is very low, so that any structure composed of it, however large, is light, in comparison to what it would be, constructed either of ivory or metal. This is of great consequence to us, as the lighter our artificial teeth are, the better, providing we have sufficient strength.

This brings us to its *solidity;* and here would appear, at first sight, to lie its weak point, instead of which we have its greatest excellence. It will not alter its shape at any temperature under 100, whilst at 20 degrees higher temperature, it may be moulded to the most intricate shape, with the greatest facility. At a low temperature it is hard and unyielding, and at the usual temperature of the mouth, it is sufficiently solid to retain its shape under any extent of pressure, and yet too soft to injure the structures on which it presses. Its great elasticity, too, allows it to yield to resistance, only to return to its exact form again when the resistance is removed. It will sustain great friction with but slight effect, we may almost say, with impunity, although, at the temperature of the mouth, the fine filaments on the surface become slightly raised by friction. I shall presently show my plan for obviating this, which, in some cases, would be a defect. I think it right to mention all its defects as well as good properties, that the knowledge of them may assist my fellow-laborers in their future experiments on this substance when used in the mouth.

Its *durability*, under the circumstances in which we place it, is very great. It is soluble in scarcely anything but strong spirits, as ether, naptha, or chloroform. It is quite insensible to the effect of acids, and the strongest alkalies have no more effect. It is quite impervious to that great solvent, water ; and, although I have had it in the mouth, where heat and moisture combine under the most favorable circumstances for decomposition, for years, I have not found the least sign of decay.

*Color.*—If the white gutta percha be taken for the base, it can be colored to the greatest nicety of tint. But here is another defect, and, I think, its greatest; light affects it, and I do not think any color, unless brown, perhaps, will remain long uninjured, if subjected to light, as, like caoutchouc, light has power of turning it brown, the color that is seen in general use. In the mouth, this does not happen to any extent, as mostly the mouth is dark, and those parts forming the gums, I shall show, should be covered with certain substances to protect them.

*Fine Texture.*—This is a quality pre-eminently possessed by this substance, and one that is of the greatest consequence to us. I am told that, since its introduction, it has been used by seal engravers, to try their impressions on, in preference to sealing-wax; as it gives a finer impression. I have a piece that I moulded to the side of a shell, that has the prismatic colors transferred to it, which I think as great a proof as can be given of its capabilities in this respect. In use in the mouth, the impression is far more beautiful than in wax, the slightest peculiarity of texture in the mucous membrane being represented.

I shall now proceed to give a detailed account of my plan for availing ourselves of the advantages of this material. I shall follow the same plan here that I pursued in my discussion of the artificial teeth now in use—give the process of manufacture, stage by stage, until we have the complete artificial teeth. I shall then show the method of adapting them to the mouth, *after they are finished*, and how we can regain any certainty that we have lost in manufacture, and, in conclusion, say a few words in comparison of the different methods.

First, I take the best model that I can obtain in wax, in the usual way, and prepare a plaster cast from it, in which I do not attempt to rectify any defects, as that can be better done in a future process. This plaster cast I render as hard as possible, with resin and wax in equal proportions, which renders the plaster impervious to water, and enables me to immerse it in cold water with impunity.

This being completed, I construct a gold wire frame in such a manner as to support the different artificial teeth firmly in their places, and, likewise, give strength to those parts where the gutta percha is necessarily thin, as behind the front teeth in the lower jaw. This frame I firmly solder together, rendering it alone as strong as the complete structures on the old plan; but I construct it in such a manner that, in the finished teeth, it is imbedded in the centre of the gutta percha, and no part of it allowed to touch either the gums or remaining teeth, its only object being to give firmness and strength, and to attach the teeth.

I then proceed to mould gutta percha around this frame, on the plaster model, in such a manner as to produce the shape required for replacing all the deficient structures of the mouth, placing the mineral teeth already prepared on the pins fixed for their reception, and moulding the gutta percha around them, so as to replace the sockets and gum originally existing around the natural teeth. This structure may then be finished to such a nicety as to represent accurately every peculiarity of surface.

Then I place the work in the mouth at a temperature just sufficiently high to enable the gutta percha to be moulded to the mouth itself, and thus it receives the shape it is to retain until it is quite finished.

I then remove the mineral teeth, leaving the sockets that receive them empty, and the gutta percha on all sides apparent, preparatory to my last process; this being to cover all the surfaces that come in contact with friction, either from the tongue or cheeks, with a strong plate of gold, which may be obtained by the electrotype process, being particular not to have any gold in such a position that it can possibly touch the soft parts under, in pressure, during the action of the mouth, but allowing it to dip into every socket for the mineral teeth, thereby giving them great strength. The teeth are again replaced, and fastened on the gold pins in their sockets, and all that portion of the gutta percha that represents the gum I either color, by means of pigments, or enamel the surface of the gold which has been allowed to pass over it. This completes my artificial teeth, without one destructible or defective material being used, and yet without subjecting the gums to any hard, unyielding substance.

I then immerse them in water, at about 120 or 130 degrees, which accomplishes two objects—1st, removes any unpleasant sensation from the cold metal; and 2d, enables the gutta percha to intimately adapt itself to its resting-place, whilst the mouth is in action for the first few minutes, and so to receive from the mouth itself the form that it is to retain, and thus rectify any imperfection in the plaster cast or wax impression.

In conclusion, I have to compare the old system with the one I advocate. First, in the latter we rectify all uncertainty that existed in the former as to fit; next, we remove the pressure of hard substances, and equalize the pressure on the parts beneath; then we are able, from the exact fit obtained, to dispense with any fastening whatever around the remaining teeth—even in whole sets, the spiral springs are not required. I have many whole sets on this principle, now in use, that are more firm without them than they have ever been with them on the old principle. The irregularities of the remaining teeth, that stood so much in our way, are of great use to us in the new system, as they tend to support and steady the artificial structure that we have been enabled to mould directly to them, without having recourse to those duplicate impressions so incompatible with certainty. In fact, we take nature's own plan in rectifying defects—the surrounding parts mould the new structures to suit themselves; while, in the old system, any alteration or allowance that took place after the artificial teeth were in the mouth, was affected by the new teeth on the mouth, instead of the mouth on the teeth; and I need not say which is the better.

For durability, I am sure it can compare with the old plan; and all those perplexing little accidents, so common in the best work now, will be prevented by the extra strength obtained, and the support given to the teeth by the new sockets. What I have shown will, I think, be apparent to all who wear artificial teeth, and even to those who are unaccustomed to their use. Of one thing I am confident—that none of my professional brethren will gainsay one word that I have uttered here, and that this method will be adopted in all those cases adapted to its use, which are innumerable.

I find, since writing the above, that some dentists have given an unfair report to their patients of my plan. Such a proceeding scarcely deserves comment. Of all new plans, jealousies will occur; the majority, however, have given me a favorable decision. To some I have granted licenses, and done cases for others, that they might test it more fully. I shall still be happy, in any way, to further their views, if practicable. Should the public ear be abused by unfavorable reports of my plan, I can only say, "See it and judge," which can at any time be done, without fee, by granting me the favor of a visit.

